# Robotic Intracorporeal Ileal Conduit Formation: Initial Experience from a Single UK Centre

**DOI:** 10.1155/2013/642836

**Published:** 2013-09-01

**Authors:** Conrad V. Bishop, Nikhil Vasdev, Gregory Boustead, James M. Adshead

**Affiliations:** Hertfordshire and South Bedfordshire Urological Cancer Centre, Lister Hospital, Stevenage SG14AB, UK

## Abstract

*Objectives*. To describe our technique of robotic intracorporeal ileal conduit formation (RICIC) during robotic-assisted radical cystectomy (RARC). To report our initial results of this new procedure. *Patients and Methods*. Seven male and one female patients underwent RARC with RICIC over a six-month period. Demographic, operative, and outcome data was collected prospectively. Median patient age was 75 years (range 62–78 years). Median followup was 9 months (range 7–14 months). *Results*. RARC with RICIC was performed successfully in all eight patients. The median total operating time was 360 minutes (range 310–440 minutes) with a median blood loss of 225 mL (range 50–1000 mL). The median length of stay was nine days (range 6–34 days). Four patients (50%) were discharged within seven days. Four patients (50%) experienced one or more complications. This included two Clavien I complications, two Clavien II complications, and two Clavien III complications. Two patients (25%) required transfusion of two units each. To date, there have been no complications associated with the ileal conduit. *Conclusion*. Whilst being technically challenging, this procedure is safe, feasible, and reproducible. Patients who avoid complication show potential for rapid recovery and early discharge.

## 1. Introduction

Advances in robotic surgery over the last decade have led to increasingly more complex procedures being undertaken. Robotic-assisted radical cystectomy (RARC) with extracorporeal diversion has become an established alternative to open radical cystectomy (ORC) in many specialist centres worldwide [1–6]. More recently, we have seen the evolution of robotic intracorporeal ileal conduit (RICIC) formation, although this is still relatively in its infancy and is limited to a handful of centres [7–10]. To the best of our knowledge, at the time of writing there are only four surgeons at four separate institutions performing RICIC in the United Kingdom. This is the first report of RICIC results in the UK.

Our robotic surgery program at the Hertfordshire and South Bedfordshire Urological Cancer Centre began in 2008, beginning with robotic-assisted radical prostatectomy (RARP). RARC was introduced in 2010 and our first RARC with RICIC was performed in January 2012. In this paper, we describe our technique of RICIC and report our initial results.

## 2. Patients and Methods

Patient selection for RARC with RICIC followed our same guidelines for RARC with extracorporeal diversion. In general, we avoid patients with previous major abdominal surgery as for any procedure requiring pneumoperitoneum. We also avoid patients who may not tolerate prolonged surgery in steep trendelenburg such as those with obesity, cardiac disease, poor lung function, or peripheral vascular disease. We also limit the procedure to patients with CIS, T1, or T2 disease. Patients with T3b or T4 disease are more technically challenging and often require a large incision to remove the specimen; we feel there is likely little or no benefit in attempting these cases robotically.

The workload of the cystectomy and pelvic lymph node dissection was shared equally between four surgeons (CVB, NV, GB, and JA). All the subsequent RICICs in this series were performed by all 4 surgeons. A prospective database was maintained for all patients undergoing RARC with RICIC. This included demographic, clinical, operative, pathological, and outcome data.

**Figure 1:**
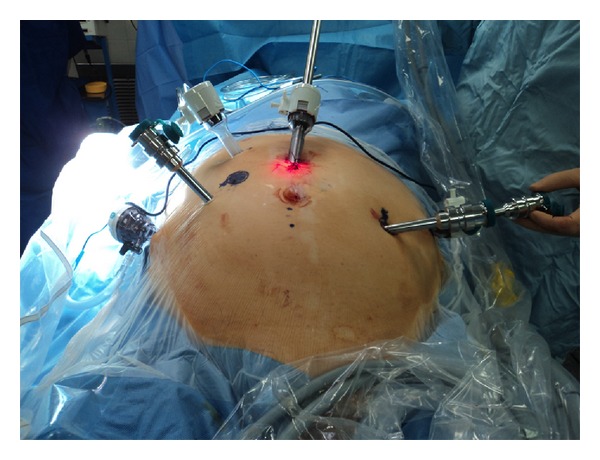
Port placement for RARC with RICIC. The port for the fourth arm is “piggy backed” through a 12 mm instrument port on the patient's left side (partially obscured by the third arm port). The fourth arm and it is port can be undocked to allow access for a stapling device for the bowel anastomosis. The camera port needs to be at least 5 cm above the umbilicus to allow room for bowel and ureteric anastomoses. Ideally, the port for the right robotic arm is placed at the site for the intended stoma; however, this was too medial to be of use in this patient.

Our technique begins with a standard robotic cystectomy and extended pelvic lymph node dissection. For this part, the patient is in a low lithotomy position in a steep 30-degree trendelenburg tilt. Our six-port placement is shown in Figure 1. The ports for RICIC are placed more cephalad than a standard RARC to allow more room for the bowel and ureteric anastomoses. Ideally, the port for the right robotic arm is placed through the marked stoma site. This prevents an extra incision; however, it may not be feasible in patients requiring a more medial stoma—a medial placement will lead to clashing of the right arm with the camera. We “piggy back” the port for the fourth robotic arm through a 12 mm port on the patient's left side. The fourth arm can then be undocked and a stapling device may be passed through the 12 mm port for the bowel anastomosis. This avoids a further 12 mm port being utilized, such as the suprapubic port described in Guru's marionette technique [7].

Once the cystectomy and extended pelvic node dissection is complete, the robot is briefly undocked and the trendelenburg tilt is reduced to 10 degrees. This allows for easier handling of the bowel and has the added benefit of restoring blood flow to the legs.

A window is made under the sigmoid mesocolon to allow the transposition of the left ureter to the right side. For this maneuver, the assistant places an endoscopic grasper at the base of the right mesocolon. The sigmoid is then flipped over to the right side and the window is dissected from the left. The end of the assistant's grasper can be identified through the mesocolon by gentle agitation from the assistant, from which it may be dissected to complete the window safely, avoiding vascular injury. The assistant may then advance the grasper through the window and pull the ureter through to the right side.

For handling the bowel, we use the robotic Cadiere forceps, which are less traumatic than the Prograsp or Maryland forceps. The caecum is identified, and the ileum is brought into the vacant pelvis. A Vicryl suture is placed approximately 20 cm proximal to the ileocaecal junction to mark the stoma end of the conduit, preserving the terminal ileum. A 12–20 cm length of ileum is then resected depending on the patient's body habitus. A 15 cm length of suture material may be introduced to help measure the intended length of ileum for the conduit. A 45 mm vascular stapling device is used to resect the ileum. This may be introduced through either the assistant ports or the 12 mm port on the patient's left side used for the fourth arm. The stapler should be angled in a fashion that allows for transection of the bowel and mesentery at right angles to ensure that vascularity to the conduit is maintained. Care should also be taken to ensure a broad base at the root of the mesentery to maintain vascularity. A side to side ileal anastomosis is then performed, this is easier done with the stapler introduced via the left-sided port.

The distal ends of the ureters are joined by a single Vicryl suture, this is then retracted by the fourth arm towards the anterior abdominal wall to bring them into clear space. The ends are then spatulated in preparation for a Wallace “66” anastomosis. The back wall of the Wallace plate is secured with a continuous 4.0 V-Loc unidirectional barbed suture. No knot tying is required, speeding up the process. In a modification to the description of the marionette technique [7], the proximal end of the conduit is suspended from the anterior abdominal wall with a Vicryl suture, in close approximation to the Wallace plate. Hanging both the ureters and the conduit end into space within close approximation is the key to a straightforward ureteroileal anastomosis. The ureteroileal anastomosis is constructed with two 15 cm lengths of 4.0 V-Loc in a continuous fashion from the heel of the spatulation to the toe on each side. The right side is sutured first. After this is complete, two 6Fr stents are introduced by the assistant, which are inserted into each ureter by the robotic arms. The assistant's grasper is then passed through the conduit like an arm through a coat sleeve, where the ends of the stents can be grasped and pulled through the conduit. The left side of the ureteroileal anastomosis is then completed. The right robotic arm is undocked and the assistant makes the incision for the stoma. Under vision, the assistant insets a Babcock forceps and grasps the distal end of the conduit and retrieves it through the incision. The stoma is then sutured in the standard fashion. All patients had extended lymph node dissection performed with a median lymph node number in our series. The boundaries of the dissection were the obturator nerve medially, the genitofemoral nerve laterally, and the common iliac vessels proximally. The collapsed external iliac veins and obturator nerves were identified and avoided. The perivesical and paraprostatic lymph nodes were separately en bloc with the bladder specimen.

## 3. Results

We performed eight consecutive cases of RARC with RICIC from January 2012 to June 2012. The mean operative time was 250 minutes for robot-assisted radical cystectomy, 49 minutes for pelvic lymph node dissection, and 142 minutes for urinary diversion.

This included seven male and one female patients. Six patients had T2G3 TCC diagnosed preoperatively, two of which underwent neoadjuvant chemotherapy. Two patients had CIS and T1G3 diseases, respectively, both recurring after a six-week course of BCG. The patient age, operative details, and length of stay data are summarized in Table 1.

Of note, four patients (50%) had no complications and were discharged within seven days.

Four patients (50%) experienced at least one complication. There were six Clavien [11] graded complications; these are summarized in Table 2.

There were two Clavien 2 graded complications of note. Patient 7 had a long history of ulcerative colitis, medically treated with Mesalazine. He developed an acute episode of colitis after an ileus, which prevented him from taking his oral medication. After consultation with his gastroenterologist, he was treated with intravenous steroids for a period of seven days. This, in combination with his earlier ileus, delayed discharge until day 24.

Patient 8, who also suffered from type 2 diabetes, had been treated for an episode of bacterial conjunctivitis preoperatively with oral ciprofloxacin. He developed severe diarrhea on postoperative day four which did not settle. A faecal specimen later confirmed a diagnosis of C. difficile colitis. He was placed in isolation until his symptoms settled and faecal testing was negative. This delayed discharge until day 34.

There were two Clavien 3 graded complications in this series. Patient 5 had a straightforward operation with only 200 mL estimated blood loss. The following morning, he had abdominal pain and signs of peritonism. A check Hb returned at 6.8 g/dL despite a low output from the drain. He was taken back to theatre for exploration through a minilaparotomy incision. A small arterial bleeder was found on the staple line of the ileal anastomosis at the junction of the antimesenteric borders. This was controlled with a single Vicryl suture. He required a transfusion of 2 units of packed cells. He was discharged on day 11 and made a good recovery.

Patient 6 had a long and difficult operation (440 mins—the longest of the series). During the operation, the tissue around the right bladder pedicle was very dense without any clear tissue plane. He had more intraoperative bleeding than usual, with an estimated blood loss of 1000 mL. He received a transfusion of two units of blood perioperatively. Shortly after the case, he was noted to have a high output of what looked like dark venous blood from his drain. He remained haemodynamically stable. Given the complexity of the case, we decided to err on the side of caution and take him back to theatre. Exploration was performed with a laparoscope. There was minimal blood in the abdomen and no point of bleeding identified. No further action was taken and his drain output settled. He had an ileus postoperatively which delayed discharge until day 17. His final pathology was upgraded to T3b, with macroscopic tumour extension on the right side, explaining the operative difficulties. Fortunately, the surgical margins were clear.

There were no Clavien graded 4 or 5 complications.

There were no readmissions within 30 days.

Of note, there were no complications related to the conduit in any of the patients either acutely or to followup.

The final pathology showed CIS in one patient, T1G3 with CIS in one patient, and T2G3 in five patients, and one patient was upgraded to T3b. Margins were clear in all patients.

## 4. Discussion

**Figure 2:**
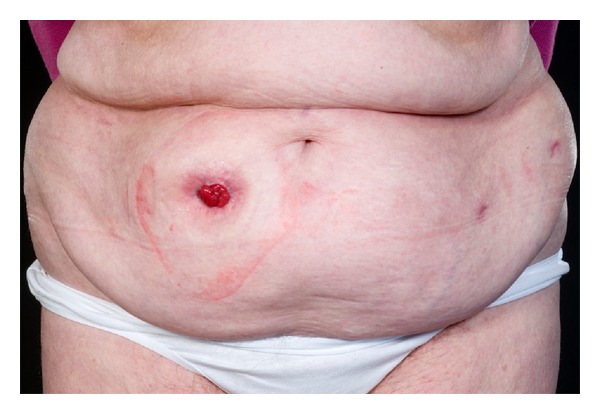
The cosmetic benefits of RARC with RICIC in a female patient two months after surgery. The specimen was retrieved through the vagina during the operation.

**Figure 3:**
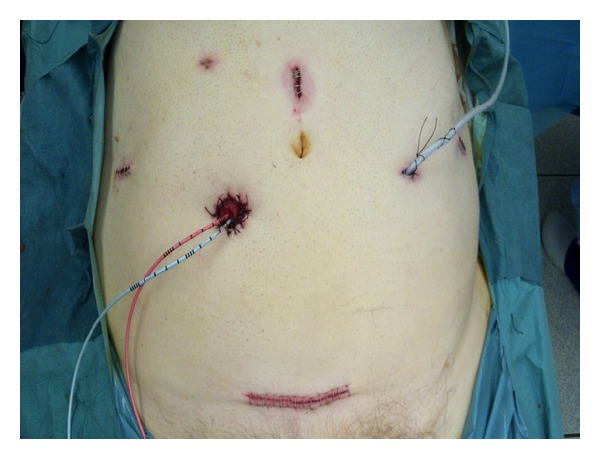
The cosmetic benefits of RARC with RICIC in a male patient immediately after surgery. The specimen was retrieved through a mini-pfannenstiel incision.

RARC has been shown in a randomized controlled study to be of benefit over ORC in selected patients with less blood loss and a shorter hospital stay [5]. To date, there is no such evidence to suggest that RARC with RICIC is superior to a standard RARC. This is partly due to the low numbers of RICIC that have been performed in comparison to RARC and ORC. Potential benefits that have been mooted include faster return of bowel function, less pain, quicker discharge and recovery, and higher patient satisfaction [8–10].  Figure 2 shows the cosmetic benefit of RICIC in a female patient.  Figure 3 shows the cosmetic appearence in a male patient following a robotic cystoprostatectomy. One of the difficulties in establishing any benefit for RICIC is that radical cystectomy is a morbid operation, performed on a patient population prone to comorbidity with a high complication rate—regardless of whether this is done open or robotically. Many of the complications are due to the cystectomy and extended pelvic node dissection, which is performed the same in both RARC and RARC with RICIC.

Our experience is that the group of patients who avoid complication do very well and likely benefit from RICIC. Four of our patients (50%) had no complication, and were discharged within a week. All had minimal analgesia requirements, early return of appetite, and bowel function and were quickly mobile. Of these patients, two were discharged on day six and two on day seven. All four patients were cleared medically for discharge 1-2 days earlier than the actual discharge, but were held back by stomal therapy. Recognizing this, we are currently negotiating for more intensive preoperative stomal therapy, with the aim of teaching patients to be independent with a stoma before the operation takes place. With this, we aim to reduce the length of stay in uncomplicated patients even further.

Four patients in this series (50%) suffered one or more complications leading to delayed discharge. In this group, we do not believe that the patients had any benefit from RICIC over a standard RARC. The difficulty remains in predicting which patients will experience a complication and have a prolonged admission.

Considering the complex nature of both the procedure and the nature of the patient population, we have been happy with our outcomes so far. Khurshid Guru from Roswell Park Cancer Institute in the USA is widely considered to be one of the world's leading RICIC surgeons. He has recently published the largest series to date (100 cases) [10].  Table 3 shows a direct head to head comparison of outcomes between Bishop and Guru. We believe that our series compares very favourably to large international series, especially considering the difference in experience.

Commencing RICIC is a daunting task given the extreme complexity of the procedure. This was made more difficult in our case given the lack of surgeons in the UK with any experience in RICIC to provide mentorship. Having said that, RICIC is only an extension of an already existing procedure which we believe can be learned by an experienced robotic surgeon in a stepwise manner. The surgeon in this series had performed over 100 robotic procedures in the previous 12 months before commencing RICIC. Experience was gained incrementally, by rehearsing subsequent steps of RICIC such as transposition of the left ureter and formation of the Wallace plate during RARC before converting to finish the conduit in an extracorporeal fashion. We believe this is a safe way for surgeons already fluent in RARC to learn RICIC in the absence of an available and qualified mentor.

In concluding, we find RICIC to be a safe, feasible, and reproducible procedure in properly selected patients. While encouraged by our early results, we eagerly await further studies to quantify any advantages over traditional RARC.

## Figures and Tables

**Table 1:** Cohort data.

Patient no.	Age (years)	Total operative time (mins)	Estimated blood loss (mL)	Length of stay (days)
(1)	62	390	100	6
(2)	67	420	550	6
(3)	76	330	50	7
(4)	78	400	250	7
(5)	77	320	200*	11
(6)	75	440	1000*	17
(7)	75	320	400	24
(8)	75	310	100	34

Median	75	360	225	9

*The denotes a transfusion of 2 units.

**Table 2:** Clavien graded complications.

Clavien grade	Complication	Management
1	Ileus (2)	Conservative
2	Ulcerative colitis (1)	Medical
	C. difficile colitis (1)	Medical
3	Postoperative bleeding (2)	Exploration
4-5	Nil	

**Table 3:** Comparison of outcomes of RICIC with Guru.

	Bishop 2013	Azzouni et al. 2013 [10]
Cases	8	100
Patient age (years, median)	75	71
Op. time (mins, median)	360	352
Blood loss (mL, median)	225	300
Transfusion rate (%)	25	10
Positive margin (%)	0	4
30-day readmission (%)	0	16
Length of stay (days, median)	9	9
Clavien 0 (%)	50	19
Clavien 1-2 (%)	50	66
Clavien 3–5 (%)	25	15
